# A Putative Zn_2_Cys_6_ Transcription Factor Is Associated With Isoprothiolane Resistance in *Magnaporthe oryzae*

**DOI:** 10.3389/fmicb.2018.02608

**Published:** 2018-10-31

**Authors:** Zuo-Qian Wang, Fan-Zhu Meng, Ming-Ming Zhang, Liang-Fen Yin, Wei-Xiao Yin, Yang Lin, Tom Hsiang, You-Liang Peng, Zong-Hua Wang, Chao-Xi Luo

**Affiliations:** ^1^Department of Plant Pathology, College of Plant Science and Technology, Huazhong Agricultural University, Wuhan, China; ^2^The Key Lab of Crop Disease Monitoring & Safety Control in Hubei Province, Huazhong Agricultural University, Wuhan, China; ^3^School of Environmental Sciences, University of Guelph, Guelph, ON, Canada; ^4^Department of Plant Pathology, College of Plant Protection, China Agricultural University, Beijing, China; ^5^College of Plant Protection, Fujian Agriculture and Forestry University, Fuzhou, China

**Keywords:** *Magnaporthe oryzae*, isoprothiolane, fungicide resistance, Zn_2_Cys_6_ transcription factor, cross resistance, CBIs

## Abstract

Isoprothiolane (IPT), a systemic fungicide, has been applied to control rice blast since the 1970s. Although resistance to IPT has been observed, the mechanism of resistance still has not been fully elucidated. In this study, nucleotide polymorphisms were detected between two IPT-resistant mutants generated in the lab, and their parental wild type isolates using a whole-genome sequencing approach. In the genomes of the two resistant mutants, single point mutations were identified in a gene encoding a Zn_2_Cys_6_ transcription factor-like protein. Notably, either knocking out the gene or replacing the wild type allele with the mutant allele (R343W) in a wild type isolate resulted in resistance to IPT, indicating that the gene is associated with IPT resistance, and thus was designated as *MoIRR* (*Magnaporthe oryzae* isoprothiolane resistance related). Along with point mutations R343W in mutant 1a_mut, and R345C in 1c_mut, a 16 bp insertion in 6c_mut was also located in the Fungal_TF_MHR domain of MoIRR, revealing that this domain may be the core element for IPT resistance. In addition, IPT-resistant mutants and transformants showed cross-resistance with iprobenfos (IBP), which was consistent with previous observations. These results indicated that MoIRR is strongly connected to resistance to choline biosynthesis inhibitor (CBI), and further work should focus on investigating downstream effects of MoIRR.

## Introduction

Rice is the most important crop worldwide, as it is the main staple food for more than half of the human population, and more than 3.5 billion people may consume rice every day. The demand for rice is growing dramatically in Asia and Africa, due to explosive population growth. However this is not matched by increasing yield, and the situation is worsened by losses to various pathogens or pests. Efficient disease control is one route to increase rice production to meet the growing demand (Seck et al., 2012; Nhamo et al., 2014).

Rice blast caused by the ascomycete fungus *Magnaporthe oryzae* is one of the most devastating diseases of rice. It leads to tremendous losses in yield and decreased quality of harvested rice, threatening world food security (Wilson and Talbot, 2009; Dean et al., 2012). Rice blast causes 10–30% losses in the field annually, but under epidemic conditions, losses are even greater (Dean et al., 2012). Furthermore, *M. oryzae* was also identified in 1985 to cause wheat blast in South America (Urashima, 1993; Malaker et al., 2016), and in 2016, wheat blast epidemics were found across 15,000 hectares in Bangladesh (Islam et al., 2016). Currently wheat blast is not only a regional disease but also a potential global crop disease (Tufan et al., 2009).

Breeding resistant cultivars is considered one of the most efficient ways to control rice blast disease. However, resistant cultivars frequently lose their effectiveness against the evolving pathogen population within a few years (Chuma et al., 2011; Dong et al., 2015). Therefore, fungicide application is still considered an efficient way to control rice blast. Isoprothiolane (Fuji-one®, diisopropyl 1, 3-dithiolan-2-ylidene-malonate, IPT) has been deployed to control rice blast since 1975. IPT significantly inhibits penetration and growth of infective hyphae, mycelial growth is inhibited completely at 20 μg/ml IPT, with emergence of infection pegs is reduced by 96% at 10 μg/ml IPT on rice leaves (Araki and Miyagi, 1976). IPT is considered a choline biosynthesis inhibitor (CBI) similar to organophosphorus fungicides because the inhibition of transmethylation from methionine into choline was observed in intact mycelia when incubated with 100 μg/ml IBP (Iprobenfos, another CBI) or 40 μg/ml IPT (Yoshida et al., 1984; Uesugi, 2001). However, the mode of action of IPT against *M. oryzae* is not fully understood. In our previous study, three IPT-resistant mutants were generated by exposing the mycelia to increasing concentrations of IPT. Transmethylation candidate genes in the biosynthesis of phosphatidylcholine, *PEAMT, CHO2*, and *OPI3* were analyzed by sequencing and evaluating expression, but no significant differences were observed between parental isolates and IPT-resistant mutants (Hu et al., 2014). With a many years intensive applications, resistance to IPT has emerged in many rice production areas (Yuan and Yang, 2003; Yuan et al., 2005; Xi et al., 2009). Timely monitoring of the dynamics of resistance is crucial for implementing scientific strategies to manage resistant populations of *M. oryzae*.

Fungicide resistance may be conferred by different mechanisms. For example in *Fusarium graminearum*, the point mutations S217L and E420K in the target gene *FgMyo1*were responsible for resistance to the fungicide phenamacril (Zhang et al., 2015). Overexpression of the *MfCYP51* gene confers resistance to DMI fungicides in *Monilinia fructicola* (Luo et al., 2008; Chen et al., 2017). Another resistance mechanism is increased fungicide efflux caused by overexpression of the ATP-binding cassette (ABC) transporter or major facilitator superfamily (MFS) encoding genes (Waard, 1997; Hayashi et al., 2001; Schoonbeek et al., 2001; Crouzet et al., 2006; Blum et al., 2010).

One approach to investigate the mechanism of fungicide resistance is molecular analysis of resistant mutants. In recent times, high throughput sequencing technology and associated bioinformatic analysis have become readily accessible and affordable (Goecks et al., 2010). In a study of resistance to bactericides in clinical bacteria, whole-genome sequencing was successfully used to identify mutations associated with resistance (Toprak et al., 2012; Farhat et al., 2013). Similarly, myosin was identified as the target of the fungicide phenamacril in *F. graminearum* by whole-genome sequencing and transcriptome analysis (Zhang et al., 2015; Zheng et al., 2015).

The purpose of this study was to identify gene mutations that might be responsible for resistance to IPT. In the current study, whole-genome sequencing combined with SNP analysis was applied to detect mutations in lab-generated resistant mutants compared with parental wild type isolates. We found mutations in a novel regulatory zinc finger protein, and confirmed its role in resistance to IPT through genetic transformation and complementation.

## Materials and methods

### Fungal strains and growth conditions

Three IPT resistant mutants and their parental isolates were used in this study. Three wild type isolates, *M. oryzae* H08-1a, H08-1c and H08-6c, were collected in 2018 from Enshi, Hubei Province, China from a field of *Oryza sativa*, and these isolates did not grow on 40 μg/ml IPT-amended PDA (Hu et al., 2014). Resistant mutants 1a_mut, 1c_mut and 6c_mut were generated in a previous study by continuously exposing parental isolates to IPT-amended PDA at concentrations ranging from 15 to 120 μg/ml (Hu et al., 2014). All single-spore strains were stored on dried colonized filter paper discs at −20°C and were recovered by placing filter paper discs on PDA followed by incubation at 27°C for 5 days in dark.

### Fungicide sensitivity assays

Sensitivity to IPT was assessed on fungicide-amended PDA at 0, 1, 5, 10, 20, and 50 μg/ml. For each strain, 5 mm diameter mycelial plugs were taken from the edge of 5-day-old colonies grown on PDA and were placed at the center of 9 cm diameter plates of PDA amended with IPT. After strains were incubated at 27°C for 12 days, colony diameters were measured from three replicate plates per concentration per isolate. Growth inhibition was calculated and regression against the logarithm of fungicide concentrations was analyzed to obtain EC_50_ values (the fungicide concentration which inhibits mycelial growth by 50%). Sensitivity to IBP was assessed on PDA amended with fungicide at 0, 10, 30, 50, 80, and 100 μg/ml.

### Evaluation of conidial production, conidial germination, mycelial growth, and pathogenicity

Isolates were grown on PDA, and hyphal plugs from actively growing margins were placed on OTA medium (40 g/L oatmeal, 150 ml/L tomato juice, and 20 g/L agar) for spore production. These cultures were incubated at 27°C under light for 5 days, 2 ml autoclaved distilled water was added, and the plate surface was gently scraped. The fluid was collected, and 0.5 ml of the hyphal-conidial suspension was spread on fresh OTA in each 9 cm diameter plate. After 36 h, aerial growth was removed by sterile wet cotton swabs, and the plate was covered with double layers of autoclaved gauze. Forty-eight hours later, conidia were harvested by flooding the colonies with 5 ml autoclaved distilled water, and gently scraping the surface of medium. The concentration of conidia in the suspensions was calculated using a haemocytometer, with three replicate readings per isolate, and adjusted to target concentrations with water. Conidial germination was observed and assessed by microscopy after 4 h on 1% agar. To evaluate the pathogenicity of wild type isolates, mutants and transformants, mycelial plugs were inoculated onto rice leaf fragments and incubated at 27°C in the dark for 24 h, and then incubated under light for 2 days. Mycelial growth on PDA was measured for daily six days post inoculation.

### Genome sequencing, assembling, and annotation

PDA plugs with mycelia were inoculated into 40 mL of potato dextrose broth (PDB) and incubated at 27°C on an orbital shaker at 150 rpm for 3 days. Genomic DNA of isolate H08-1a, H08-1c, 1a_mut, and 1c_mut was extracted by the CTAB method (Nitta et al., 1997). Genome sequencing was done on the Illumina HiSeq 4000 PE150 platform using 150 bp paired-end libraries with 500 bp inserts at Novogene Corporation (Novogene, Beijing, China). Sample H08-1a had 4 Gb raw data (targeting 100-fold coverage), while the other three strains were sequenced for 2 Gb raw data (targeting 50-fold coverage). After sequencing, read quality was assessed using the program FASTQC (www.bioinformatics.bbsrc.ac.uk/projects/fastqc/), reads were trimmed with the NGSQC Toolkit v2.3.3 (https://ccbr.github.io/Pipeliner/Tools/NGS_QC_Toolkit.html), and positions with quality score less than 30 were removed. Read sets of H08-1a or H08-1c were assembled into contigs using SOAPdenovo2 using a range of K-mer values (http://soap.genomics.org.cn/soapdenovo.html) and the genome assembly with K-mer 45 was selected because K-mer 45 provided the highest N50 (the sequence length of the shortest contig at 50% of the total genome length). Then gaps were closed with pair-end reads sets using GapCloser (Haridas et al., 2011; Dong et al., 2015). Draft genome assemblies of H08-1a and H08-1c have been deposited at the NCBI Genome Database (NKQF00000000.1 and NKQG00000000.1). Genome sequence reads data of H08-1a, H08-1c, 1a_mut, and 1c_mut were deposited at the GenBank SRA database under accession numbers SRX3336022, SRX3336023, SRX3336104, and SRX3336103. Gene predictions were done using Augustus (http://augustus.gobics.de/). Genome assembly and gene prediction completeness were assessed by BUSCO comparison to Sordariomycete dataset (https://busco.ezlab.org/). Nucleotide variations between H08-1a and 1a_mut, H08-1c and 1c_mut were analyzed using Genome Analysis Toolkit (https://software.broadinstitute.org/gatk/) with H08-1a and H08-1c as reference genomes. The published genomes of *M. oryzae* (70-15 version 8, Y34, and P131) (Dean et al., 2005; Xue et al., 2012) were downloaded from NCBI (https://www.ncbi.nlm.nih.gov/genome/genomes/62?/) and used to analyze variations between 1a_mut and corresponding genomes. Since most mutations conferring fungicide resistance are located in the upstream or coding regions of the target genes (Lucas et al., 2015), special attention was paid to these regions. Locations of polymorphisms between parental and derived resistant strains were annotated using SnpEff (Cingolani et al., 2012). Because *M. oryzae* normally grows as a haploid (Crawford et al., 1986), then within isolate polymorphism does not derive from homologous genes of paired chromosomes as would be found with diploids or dikaryons. Such variants with two type mutation codes at one locus were called ‘hemi-SNPs’ (Trick et al., 2009), and removed from the list of candidate variants. *In silico* elimination of possible sequencing errors was done by selecting possible polymorphic sites and examining each more carefully using read data to see whether potential polymorphisms were present in the raw read data of the parental strain following Nielsen et al. (2011). If such polymorphisms within the sequencing data of parental strains were found, then they were removed from the list of potential mutated sites. This manual check was to reduce the possibility of false positive mutation calls, and the check was performed for each site difference between a parental isolate and the mutant progeny. Genes functions were annotated by local blast to the gene set of published reference genome *M. oryzae* 70-15 version 8.

### Genetic transformations

Knockout transformants were generated by transforming split-marker fragments into protoplasts (Lin et al., 2011; Son et al., 2011). In brief, ~1 kb 5′ and 3′ flanking sequences of the *MoIRR* gene were amplified from genomic DNA of isolate H08-1a. Then, the 5′ flanking, 3′ flanking and a Hyg resistance cassette, which had been amplified from the vector PSKH were fused with double-joint PCR. There were 20 bp overlaps between the 5′ flanking amplicon and Hyg and between Hyg and the 3′ flanking amplicon. Finally, split-marker fragments of target genes were amplified with nested PCR primer pairs and transformed into protoplasts of H08-1a via polyethylene glycol (PEG) mediation (Figure S1A). Protoplast generation was followed the procedure described previously (Li et al., 2010). Knockout transformants were first verified with the specific primer pair IRR-UP/IRR-V and IRR-UP/Hyg-V. IRR-UP was located upstream of the split-marker fragment shown in Figure S1. IRR-V and Hyg-V were located in the *MoIRR* coding sequence and the coding sequence of the hygromycin resistance cassette, respectively. Knockout transformants were also verified by RT-PCR. Extracted RNAs were reverse transcribed into cDNA and amplified with the primer pair MoIRR_RT_F/MoIRR_RT_R. Verification data are shown in Figure S2.

Complemented transformants were obtained by random insertion of a fragment consisting of the full length *MoIRR* gene with a 1 kb upstream region and the selection marker *Npt2* gene. The fragments of *MoIRR* were amplified from H08-1a with PrimeStar HS (Takara Biomedical Technology, Beijing, China) and ligated into a pMD18-T vector with the *Npt2* gene (Takara Biomedical Technology) after nucleotide A was added by Taq polymerase (TransGen Biotech, Beijing, China). The insertion fragment was cut from the vector with *Xba*I and *Hin*dIII (Takara Biomedical Technology), and transformed into protoplasts of knockout transformant using PEG-mediated transformation.

To replace the *MoIRR* gene from 1a_mut into H08-1a, the full length *MoIRR* gene, including the promoter and 3′ flanking sequences, were cloned with PrimeStar HS (Takara Biomedical Technology) from 1a_mut genomic DNA. Double-joint PCR was applied by 20 bp overlap with the Hyg gene. Two kilobases of the 3′ flanking region were amplified from 1a_mut and were fused with Hyg by double-joint PCR. Then, two part split-marker fragments were amplified with nested PCR (Figure S1B). Finally, split-marker fragments were transferred into protoplasts of the isolate H08-1a. All primers used in this study are listed in Table S1.

### Quantitative real-time PCR

To extract RNA, mycelia were transferred into 100 ml flasks containing 40 ml PDB, and incubated at 27°C and treated with 5 μg/ml IPT 4 h before harvest. Control samples were treated at 4 h before harvest with acetone treatment as a control since acetone was the solvent used for IPT. RNA from H08-1a, 1a_mut, knockout and complemented transformants was extracted with Trizol reagent (Invitrogen, CA, USA). Residual DNA was digested with DNase I (Thermo Fisher Scientific Inc., Vilnius, Lithuania). First-strand cDNA was synthesized with a RevertAid First Strand cDNA Synthesis Kitemploying the oligo (dT) 18 primer (Thermo Fisher Scientific Inc). Expression of the *MoIRR* gene was detected by quantitative real-time PCR with the primer pair MoIRR_qF/MoIRR_qR. Real-time PCR was performed in a CFX96 Real-Time PCR detection system (Bio-Rad Laboratories, California, USA) using SYBR Green I fluorescent dye (Aidlab, Beijing, China) in 20 μl volumes with 1 μl cDNA and primers. All the real-time PCR experiments were performed with three independent biological repeats. The expression of the *MoIRR* gene was normalized to the expression of the β-tubulin gene, and relative gene expression was calculated with the comparative Ct (2^−ΔΔCt^) method (Wong and Medrano, 2005).

### Phylogenetic analysis

Annotated Zn_2_Cys_6_ transcription factors were collected from the NCBI protein database based on accession numbers whose functions have been reported. At the same time, three homologs showing the highest similarity to MoIRR were also selected for phylogenetic analysis. All protein sequences were aligned using ClustalW in MEGA 6 (http://www.megasoftware.net/home). Phylogenetic tree was constructed based on the Fungal_TF_MHR sequences with the neighbor-joining algorithm. Protein domain architecture analysis was performed by a Conserved Domains Database search (https://www.ncbi.nlm.nih.gov/cdd).

### Statistics

Statistical differences in the data were evaluated by one-way analysis of variance (ANOVA) along with Duncan's Multiple Range tests in SPSS for Windows Version 19.0 (SPSS Inc., Chicago, IL, USA).

## Results

### Genome sequencing, assembly, and SNP calling

**Figure 1:**
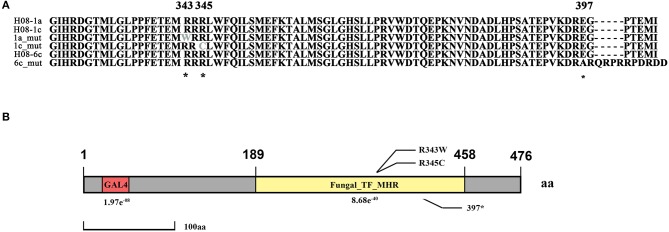
Isoprothiolane resistant mutants contain the point mutations at codon 343 and 345 of the *MoIRR* gene in *Magnaporthe oryzae*. **(A)** Alignment of amino acid sequences of MoIRR from the parental isolates H08-1a, H08-1c, and H08-6c, and resistant mutants 1a_mut, 1c_mut, and 6c_mut. The asterisks indicate the amino acid changes at the codons 343 and 345, found in 1a_mut and 1c_mut, respectively, and also the 16 bp insertion at the codon 397 in 6c_mut. *indicates mutation sites. **(B)** Schematic representation of *M. oryzae* MoIRR. R343W and R345C were two mutation sites, and position 397 was the translation frame shift caused by the 16 bp insertion. The GLA4 DNA binding domain is highlighted in red. The Fungal_TF_MHR domain (fungal transcription factor middle homology region) is highlighted in yellow.

Previously, three IPT-resistant mutants were generated locally on IPT-amended media (Hu et al., 2014). To detect sequence polymorphisms in resistant mutants, the genomes of two mutants (1a_mut and 1c_mut) and their parental isolates (H08-1a and H08-1c) were sequenced. Four Gb raw data were obtained for H08-1a, and 2Gb raw data were obtained for each of the other three strains, representing 100-fold and 50-fold coverage, respectively. From the genomes of H08-1a and H08-1c, *de novo* draft assemblies of 40.0 and 39.9 Mb were obtained, and the assembly completeness were 97.8% and 97.9%, gene completeness were 95.7 and 94.4%, respectively (Table 1). By using the Genome Analysis Toolkit (GATK), 7850 potential polymorphisms were detected between 1a_mut and the corresponding parental isolate H08-1a, and 8183 potential polymorphisms were assessed between genes of 1c_mut and its parental isolate H08-1c. In addition, polymorphisms were also detected between 1a_mut and the published *M. oryzae* genomes Y34, P131, and 70-15 (Dean et al., 2005; Xue et al., 2012) (Table 2). All mutations were annotated with SnpEff, resulting in 476 and 622 nonsynonymous variants in coding regions of 1a_mut, and 1c_mut compared with their respective parental isolates. After the “hemi-SNPs” were removed from the list of candidate variants, in total, 69 candidate variants were found in 1a_mut vs. H08-1a and 74 in 1c_mut vs. H08-1c. After additional manual check to reduce false positive SNP calls by scanning whether potential variants were present in the read data of the parental isolates, 11 candidate variants remained in 1a_mut and 12 in 1c_mut (Table 2). Among these candidates, only mutations in one gene were detected in both 1a_mut and 1c_mut (Tables S2, S3). This gene encodes a protein with the GAL4-like binuclear cluster DNA-binding domain originally found in GAL4, GAL4 is a positive regulator for the expression of galactose-induced genes in *Saccharomyces cerevisiae* (Kraulis et al., 1992), and a Fungal_TF_MHR (fungal transcription factor middle homology region) domain. The predicted coding sequences of this Zn_2_Cys_6_ transcription factor-like protein were different in three published genomes (70–15, Y34, P131). To validate the gene structure, the coding sequence was further analyzed by comparing its cDNA and gDNA sequences of H08-1a, the showing that the gene was 2,056 bp long and contained four introns and five exons with a 1,428 bp coding sequence encoding a protein with 476 amino acids (Figures S3, S4). Detected point mutations resulted in R343W and R345C amino acid changes in the Fungal_TF_MHR domain of the predicted protein in1a_mut and 1c_mut, respectively (Figures 1A, 1B). Additional Sanger sequencing of specifically amplified fragments confirmed the two point mutations found in the resistant mutants (1a_mut and 1c_mut). The Zn_2_Cys_6_ transcription factor-like protein coding gene was also sequenced in another resistant mutant, 6c_mut, and its parental isolate, H08-6c, and a 16 bp insertion was found at position 1,189 of the coding sequence in 6c_mut, resulting in a translation frame shift at codon 397 within the Fungal_TF_MHR domain (Figure 1A).

**Table 1:** Genome assembly statistics from *Magnaporthe oryzae* isolates H08-1a and H08-1c.

	**H08-1a^a^**	**H08-1c**
Number of contigs^b^	5458	5681
Assembly size (Mb)	40.0	39.9
N50 contig length (kb)	15.4	14.8
GC content (%)	50.76	50.87
Coverage (fold)	100	50
Assembly completeness^c^	97.8%	97.9%
Gene completeness	95.7%	94.4%

a *Assemblies of H08-1a and H08-1c were obtained using SOAPdenovo2*.

b *All Assembly features were calculated with NGSQC Toolkit*.

c *Genome assembly completeness and gene set prediction completeness were assessed by BUSCO comparison to Sordariomycete dataset*.

**Table 2:** Statistics on mutation detection in 1a_mut and 1c_mut of *Magnaporthe oryzae* against H08-1a, H08-1c, Y34, P131, and 70-15.

	**1a_mut vs. H08-1a**	**1c_mut vs. H08-1c**	**1a_mut vs. Y34^d^**	**1a_mut vs. P131**	**1a_mut vs.70-15**
Number of variants	10,059	10,476	38,687	37,632	29,712
Number of SNPs^a^	7,850	8,183	27,766	26,730	22,826
Missense variants	476	622	4,885	4,782	3,912
Upstream variants	2,077	2,196	44,672	41,416	44,243
Candidate variants^b^	69	74	NC^c^	NC	NC
Reliable variant	11	12	NC	NC	NC

a *All SNPs were annotated with SnpEff, and number of variants, number of SNPs, missense variants and upstream variants were calculated*.

b *Candidate variants refer to SNPs after removing hemi-SNPs*.

c *NC: not calculated*.

d Y34, P131, and 70-15 are published genomes of M. oryzae, and downloaded from NCBI (https://www.ncbi.nlm.nih.gov/genome/genomes/62?/)

### The *MoIRR* gene is responsible for resistance to isoprothiolane

**Figure 2:**
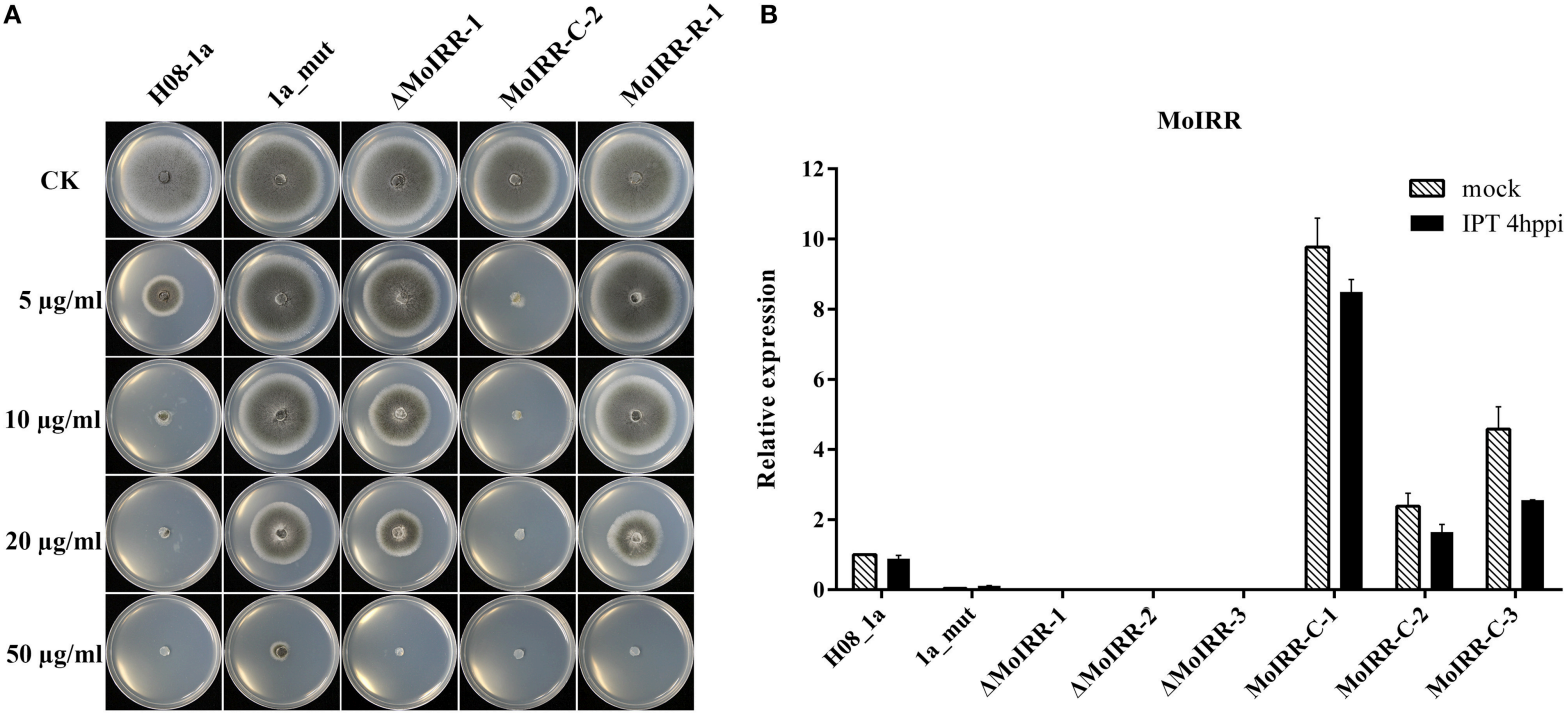
*MoIRR* gene is responsible for resistance to isoprothiolane. **(A)** Growth of *MoIRR* knockout transformant ΔMoIRR-1, replacement transformant MoIRR-R-1 and complemented transformant MoIRR-C-2 on isoprothiolane amended PDA. The mycelial plugs taken from the edge of a 5dpi colony of isolates or mutants and grew at 27°C for 6 days on the PDA plates, which were amended with IPT at 0, 5, 10, 20, and 50 μg/ml. **(B)** Expression level of *MoIRR* knockout transformants and complemented transformants was assessed with real-time PCR. RNAs were extracted from IPT or acetone treated samples, and beta-tubulin gene was used as reference.

To analyze the role of the Zn_2_Cys_6_ transcription factor-like protein encoding gene (MGG_04843), knockout transformants were generated from the parental isolate H08-1a. Knockout transformants were confirmed by specific PCR and RT-PCR (Figures S2A, S2B). All three transformants showed decreased sensitivity to IPT compared to the parental isolate H08-1a (Figure 2A; Table 3). Therefore, the Zn_2_Cys_6_ transcription factor-like protein coding gene was designated *MoIRR* (*Magnaporthe oryzae* isoprothiolane resistance related). The sensitivity of *MoIRR* knockout transformants (ΔMoIRRs) to IPT was evaluated based on mycelial growth on the IPT-amended media at 27°C. Similar to resistant mutant 1a_mut, all three *MoIRR* knockout transformants could grow on 20 μg/ml IPT-amended PDA, and EC_50_ values for IPT were almost four-fold higher than that of the parental isolate H08-1a (Figure 2A; Table 3). For complementation, the wild type *MoIRR* gene including 1 kb of the upstream region was randomly integrated into the knockout transformant ΔMoIRR-1. As expected, all three complemented transformants (MoIRR-Cs) expressed the *MoIRR* gene and were sensitive to IPT (Figures 2A,B). MoIRR-C-2 showed EC_50_ of 1.27 μg/ml, which were even lower than that of the parental isolate H08-1a (Table 3). EC_50_ values of knockout transformants were not significantly different from the resistant mutants (*P* = 0.898), giving strong evidence that the *MoIRR* gene plays a negative regulatory role in IPT resistance in *M. oryzae*.

**Table 3:** Sensitivity of wild-type, mutants and different transformants (*MoIRR* knockout transformants, complemented transformants, and replacement transformants) to IPT and IBP.

**Isolate**	**IPT EC_50_ (μg/ml)^d^**	**IBP EC_50_ (μg/ml)**
H08-1a	4.72	25.64
H08-1c	4.10	30.85
H08-6c	5.42	26.76
**Wild type isolates**	**4.750** ±**0.7a**	**27.75** ±**2.7a**
1a_mut	19.23	72.54
1c_mut	16.78	69.11
6c_mut	17.35	62.10
**Resistant mutants**	**17.79** ±**1.3b**	**67.92** ±**5.3b**
ΔMoIRR-1^a^	17.93	66.92
ΔMoIRR-2	17.74	64.67
ΔMoIRR-3	17.38	60.69
**Knockouttransformants**	**17.68** ±**0.3b**	**64.09** ±**3.2b**
MoIRR-C-1^b^	9.51	45.63
MoIRR-C-2	1.27	20.64
MoIRR-C-3	8.92	31.99
**Complemented transformants**	**6.57** ±**4.6a**	**32.75** ±**12.5a**
MoIRR-R-1^c^	16.24	61.35
MoIRR-R-2	12.98	57.59
MoIRR-R-3	12.05	57.27
**Replacement transformants**	**13.76** ±**2.2b**	**58.7** ±**2.3b**

a *ΔMoIRR-1,−2,−3 are the knockout transformants generated by transforming split-marker fragments to knockout MoIRR gene in parental isolate H08-1a*.

b *MoIRR-C-1,−2,−3 are the complemented transformants achieved by introducing MoIRR gene with 1 kb upstream region into knockout transformant ΔMoIRR-1*.

c *MoIRR-R-1,−2,−3 are the replacement transformants in which original MoIRR allele in isolate H08-1a was replaced with the MoIRR allele from the resistant mutant 1a_mut*.

d *Mean ± S.D (standard deviation of mean). Different letters within a column were calculated based on the group of knockout, complemented, and replacement transformants with Duncan test analysis indicate statistically significant differences (P = 0.05)*.

*The bold values indicate mean value of group respectively*.

### The Fungal_TF_MHR domain of the MoIRR was the core element associated with IPT resistance in *M. oryzae*

**Figure 3:**
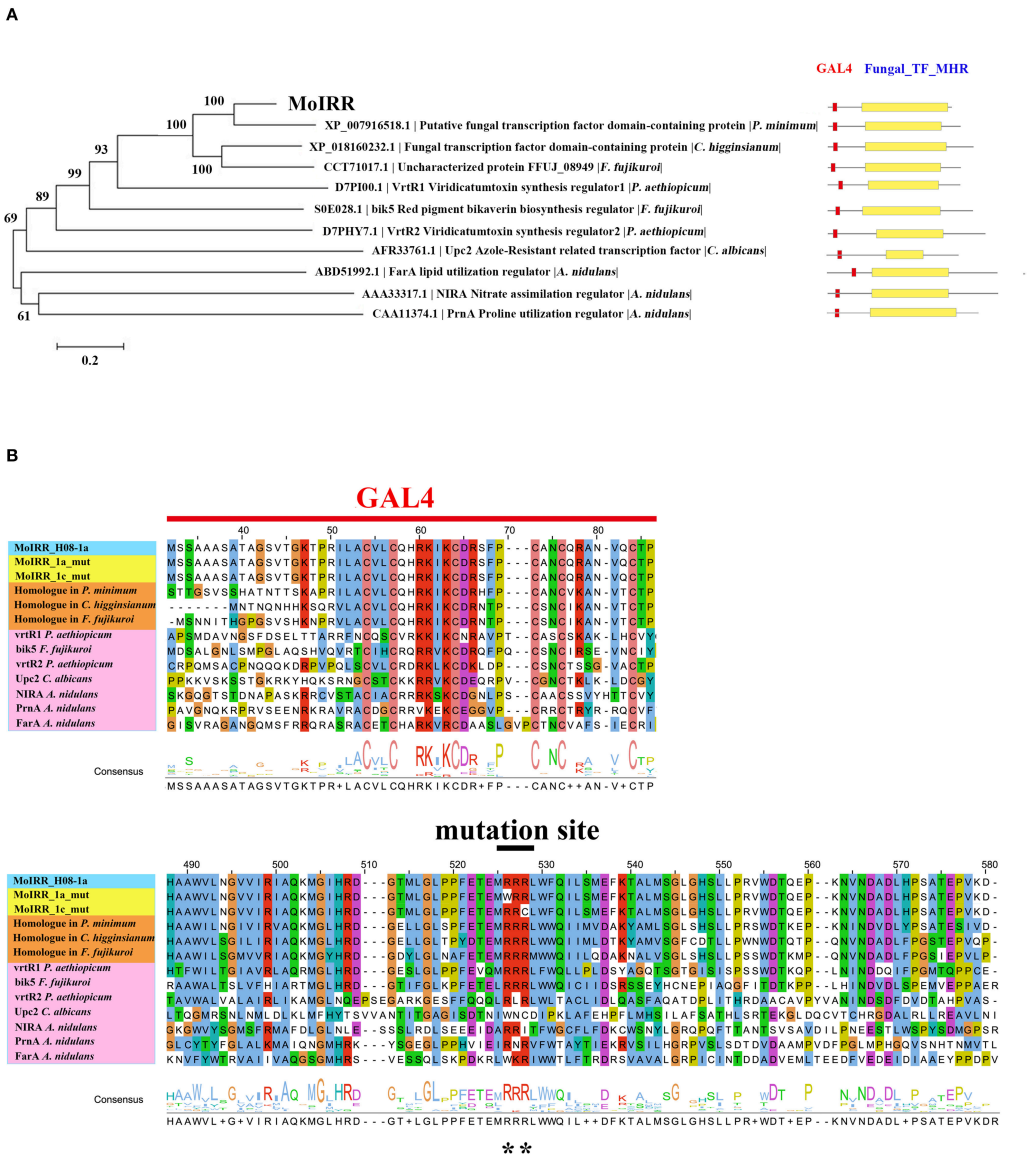
Phylogenetic tree of MoIRR homologous proteins. **(A)** The sequences of proteins in phylogenetic tree were retrieved from the NCBI database. Domains were aligned with ClustalW, and the tree was constructed with the neighbor-joining method. Scale bar, 0.2 substitutions per site. Two domains from the Zn_2_Cys_6_ proteins are indicated as red (GAL4) and yellow (Fungal TF_MHR) boxes. **(B)** Amino acid sequence alignment was constructed with ClustalW, and consensus sequences were marked with different color. Reported Zn_2_Cys_6_ proteins were highlighted with pink, three homologous proteins of MoIRR were highlighted with orange, and two resistant mutant proteins were highlighted with yellow. Graph shows part of amino acid sequence alignment. **indicates mutation sites.

Zn_2_Cys_6_ transcription factors are a subcategory of zinc finger proteins unique to fungi. They usually contain Cys-X_2_-Cys-X_6_-Cys-X_5_-12-Cys-X_2_-Cys-X_6_-9-Cys (C6 domain) with two zinc ions as a binding domain and a regulatory domain (referred to as a TF domain) (Chang and Ehrlich, 2013). To assess which type of Zn_2_Cys_6_ transcription factors MoIRR may belong to, phylogenetic trees were constructed with homologous proteins from other fungi and seven reported Zn_2_Cys_6_ transcription factors. MoIRR had the highest similarity with homologous proteins from *Phaeoacremonium minimum, Colletotrichum higginsianum*, or *Fusarium fujikuroi*, but the functions were annotated as unknown (Figure 3A). As expected, all homologous proteins of MoIRR contained a DNA-binding domain which is required for the function of transcriptional regulators (Figures 3A,B). To further confirm whether mutations in the Fungal_TF_MHR domain of the MoIRR were responsible for the resistance to IPT, the wild type *MoIRR* gene in H08-1a was replaced with the mutated *MoIRR* allele (R343W) of 1a_mut. Replacement transformants (MoIRR-Rs) were confirmed by sequence analysis. The sensitivity of *MoIRR* replacement transformants to IPT was evaluated based on mycelial growth rates on IPT-amended media. The results showed that the *MoIRR* replacement transformants, similar to knockout transformants, could grow on PDA amended with 20 μg/ml IPT (Figure 2A). The EC_50_ values for three replacement transformants were 16.2, 13.0, and 12.1 μg/ml, which were not significantly different from the resistant mutant 1a_mut (Table 3). All the *MoIRR* replacement transformants had reduced sensitivity to IPT, implying that the point mutation leading to R343W in the *MoIRR* gene was responsible for the resistance to IPT in resistant mutant 1a_mut (Figure 2A; Table 3). Considering that the point mutation leading to R345C in 1c_mut and the insertion in 6c_mut were located in the Fungal_TF_MHR domain, this domain should be the core element associated with IPT resistance in *M. oryzae*. These point mutations causing amino acid changes in 1a_mut and 1c_mut were both located at the RRR motif (Figure 3B), indicating that the RRR motif was essential for IPT resistance.

### Resistant mutants and transformants have no significant fitness penalty

**Figure 4:**
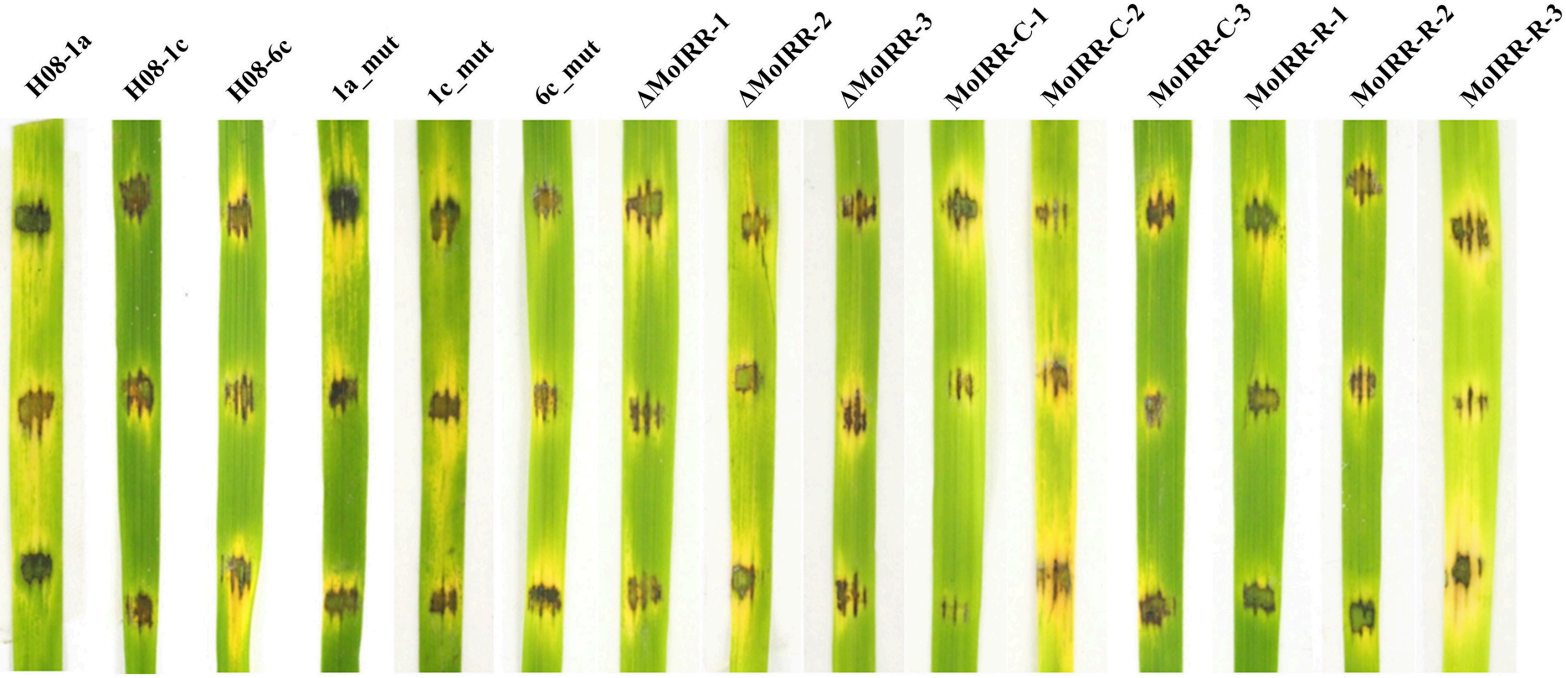
Pathogenicity assays for wild-type isolates, resistant mutants, knockout transformants, complemented transformants, and replacement transformants. Pathogenicity of wild type isolates, mutants and transformants was evaluated by inoculating mycelial plugs of individual strain onto rice leaf fragments and incubated at 27°C in the dark for 24 h, and then incubated under light for 2 days.

To evaluate whether fitness penalties occurred in resistant mutants or transformants, colony growth, conidial production, conidial germination and pathogenicity were assessed. No significant differences for either mycelial growth, conidial production or conidial germination among wild type isolates, mutants or transformants were observed (Table 4). Furthermore, all resistant mutants and transformants showed similar pathogenicity as the wild type isolates (Figure 4). These results indicated that no significant fitness penalties were observed in resistant mutants and transformants compared to sensitive wild type isolates, suggesting that MoIRR is not essential for survival.

**Table 4:** Growth rate and fitness parameters of wild-type, mutants and different transformants (*MoIRR* knockout transformants, complemented transformants, and replacement transformants) to IPT and IBP.

	**Fitness parameter**^**e**^		
**Isolate**	**Mycelial growth (mm/day)**	**Conidiation^d^ (10^5^/cm^2^)**	**Conidial germination (%)**
H08-1a	6.53 ± 0.2	3.33 ± 1.7	97.11 ± 1.2
H08-1c	7.08 ± 0.3	1.94 ± 1.1	90.44 ± 6.1
H08-6c	6.85 ± 0.2	2.55 ± 0.6	99.44 ± 0.5
**Wild type isolates**	**6.82** ±**0.3a**	**2.6** ±**0.7a**	**95.67** ±**5.2a**
1a_mut	6.92 ± 0.1	7.48 ± 2.8	96.67 ± 1.6
1c_mut	6.92 ± 0.1	1.61 ± 0.8	93.89 ± 1.6
6c_mut	6.63 ± 0.3	2.30 ± 0.9	99.00 ± 1.0
**Resistant mutants**	**6.8** ±**2.2a**	**3.8** ±**3.2a**	**96.52** ±**2.6a**
ΔMoIRR-1^a^	6.74 ± 0.3	5.33 ± 1.3	96.78 ± 2.4
ΔMoIRR-2	6.97 ± 0.2	2.63 ± 1.1	97.33 ± 1.8
ΔMoIRR-3	6.75 ± 0.2	3.40 ± 2.4	97.33 ± 2.5
**Knockout transformants**	**6.82** ±**0.1a**	**3.8** ±**1.4a**	**97.15** ±**2.2a**
MoIRR-C-1^b^	6.76 ± 0.1	1.44 ± 0.7	98.78 ± 1.4
MoIRR-C-2	6.79 ± 0.3	2.06 ± 0.8	98.33 ± 1.3
MoIRR-C-3	6.76 ± 0.2	5.43 ± 2.0	97.44 ± 1.0
**Complemented transformants**	**6.77** ±**0.1a**	**3.0** ±**2.1a**	**98.19** ±**1.7a**
MoIRR-R-1^c^	6.63 ± 0.1	3.51 ± 2.2	95.78 ± 1.4
MoIRR-R-2	6.43 ± 0.1	1.76 ± 0.9	97.67 ± 1.3
MoIRR-R-3	6.49 ± 0.1	1.55 ± 0.8	96.44 ± 1.5
**Replacement transformants**	**6.51** ±**0.1a**	**2.3** ±**1.1a**	**96.63** ±**1.6a**

a *ΔMoIRR-1,−2,−3 are the knockout transformants generated by transforming split-marker fragments to knockout MoIRR gene in parental isolate H08-1a*.

b *MoIRR-C-1,−2,−3 are the complemented transformants achieved by introducing MoIRR gene with 1 kb upstream region into knockout transformant ΔMoIRR-1*.

c *MoIRR-R-1,−2,−3 are the replacement transformants in which original MoIRR allele in isolate H08-1a was replaced with the MoIRR allele from resistant mutant 1a_mut*.

d *Conidia were generated on OTA media after 48 h of growth*.

e *Mean± S.D (standard deviation of mean). Different letters within a column were calculated based on the group of knockout, complemented, and replacement transformants with Duncan test analysis indicate statistically significant differences (P = 0.05)*.

*The bold values indicate mean value of group respectively*.

### IPT-resistant strains showed cross-resistance to IBP

**Figure 5:**
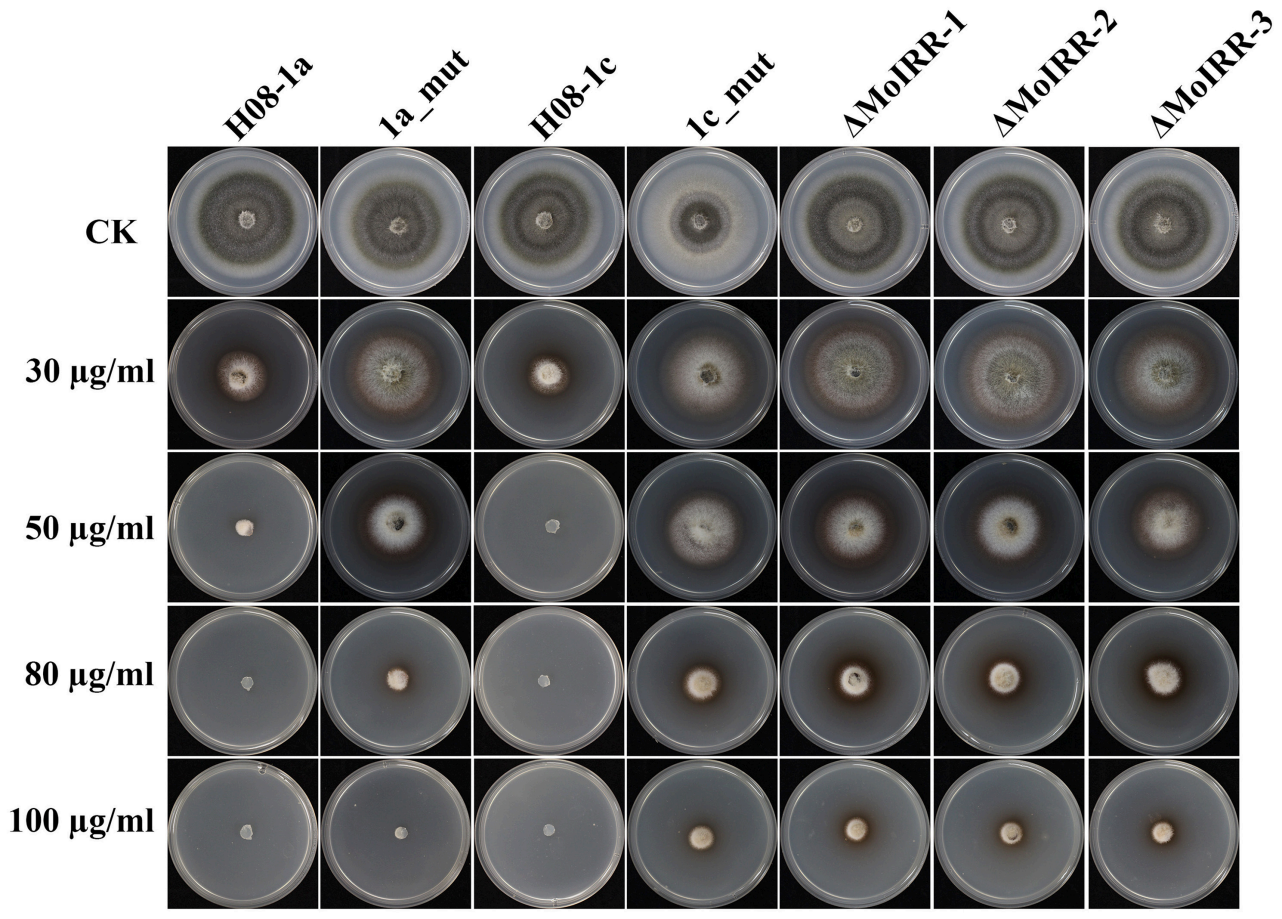
IPT resistant mutants and MoIRR knockout transformants showed cross-resistance to IBP. Growth of resistant mutants, *MoIRR* knockout transformants and parental isolates on ipobenfos amended PDA. The mycelial plugs taken from the edge of a 5 dpi colony of isolates or mutants and grew at 27°C for 6 days on the PDA plates, which were amended with IBP at 0, 30, 50, 80, and 100 μg/ml.

To evaluate whether IPT-resistant strains showed cross-resistance between the CBI's, IPT, and IBP, the sensitivity of IPT-resistant mutants, MoIRR knockout transformants and replacement transformants was assessed on IBP-amended media. We were unable to obtain technical grade IBP, and the IBP formulation used in this study also contained tricyclazole (Iprobenfos (13.3%) + Tricyclazole (6.7%), 20% W.P.). Although tricyclazole showed some inhibition of mycelial growth, no difference was observed between resistant strains and wild type isolates, indicating no cross resistance between IPT and tricyclazole (*P* = 0.327) (Table S4). Interestingly, The IBP EC_50_ values of resistant mutants, knockout transformants and replacement transformants to IBP were significantly greater than those of wild type parental isolates and complemented transformants (Table 3; Figure 5), indicating the presence of cross-resistance between IPT and IBP. Since IPT and IBP have been described to inhibit transmethylation in the phosphatidylcholine (PC) biosynthesis pathway, the expression profile of methyltransferases in PC biosynthesis pathways such as PEAMT in Kennedy's pathway and CHO2 and OPI3 in the Bremer-Greenberg pathway was investigated by real-time PCR as in a previous study (Hu et al., 2014). No significant differences were observed between resistant mutants (1a_mut) and the parental wild type isolate (H08-1a), indicating that MoIRR is likely not involved in regulating the biosynthesis of phosphatidylcholine.

## Discussion

Since IPT was developed decades ago, it has been widely used for rice blast control; resistance to this chemical is emerging (Yuan and Yang, 2003; Yuan et al., 2005; Xi et al., 2009), but the mechanism of resistance is still not known. During previous studies using resistant mutants, we did not find a gene related to resistance (Hu et al., 2014). There are similarities between organophosphorus thiolate (PTL) fungicides and IPT, particularly because cross-resistance between IPT and phosphorothiolates and negative cross-resistance between IPT and phosphoramidates have been observed (Katagiri, 1977). With ^13^C labeling, transmethylation yielding choline was found to be inhibited by PTL fungicides and IPT (Yoshida et al., 1984). Thus, IPT was thought to be a choline biosynthesis inhibitor similar to PTL fungicides. There are two main pathways for choline biosynthesis, and the Kennedy's pathway is the principal route of phosphatidyl-choline biosynthesis in *M. oryzae* (Yoshida et al., 1984). Thus, we sequenced *PEAMT, CHO2*, and *OPI3* from resistant mutants and their parental wild type isolates (Hu et al., 2014). These genes are responsible for transmethylation in choline biosynthesis, but their sequences and expression levels showed no significant differences between resistant mutants and their corresponding parental isolates. Those results suggested that the mechanism of resistance to IPT was not conferred by the variation of transmethylation genes in choline biosynthesis. In the current study, newer methods were used to further elucidate the molecular mechanism of IPT resistance in *M. oryzae*, and sequence variations in the *MoIRR* gene including point mutations or insertions were found to be associated with IPT resistance.

Through genomic comparisons, we found 69 candidate polymorphisms from 1a_mut compared to its parental isolate, and 75 in 1c_mut compared to its parent, respectively. However, after manually checking with SNP calling against the parental wild type H08-1a genome, there were 11 or 12 candidates remaining, indicating false-positive SNP calls that could be found even after variant quality score recalibration (a method to reduce false-positive calls) was used. In future studies, to reduce the number of false-positive SNP calls, the sample number and depth of sequencing can be increased or other software can be used.

Transcription factors (TFs) are proteins regulating gene expression by binding to specific DNA sequence in the promoter region. Zinc finger TFs are one of the largest groups of transcriptional regulators, many of which have been characterized (Beri et al., 1987; Johnston, 1987; Andrianopoulos and Hynes, 1990; Burger et al., 1991; Suárez et al., 1995; Todd and Andrianopoulos, 1997; Todd et al., 1997; Kim et al., 2006; Lu et al., 2014). Most TFs are involved in primary or secondary metabolism (Burger et al., 1991; Kim et al., 2006), along with stress responses and pleiotropic drug resistance (Hellauer et al., 2002). Zinc finger family proteins have been divided into three main classes according to the number and order of cysteine residues: Cys_2_His_2_ proteins, Cys_4_ zinc fingers proteins and Zn_2_Cys_6_ proteins. Zn_2_Cys_6_ transcription factors are unique in the fungal regulation network and some of them, such as virulence-required TFs GPF1 and CNF2, have been functionally analyzed by genetic transformation in *M. oryzae* (Lu et al., 2014). Attributes such as growth, asexual development and infection-related processes, pathogenicity, and tolerance of abiotic stresses have been tested previously. For MGG_04843 (MoIRR), no significant differences were observed between deletion transformants and wild type isolates (Lu et al., 2014). Similarly, our gene-targeted knockout transformants did not show significant differences in mycelial growth, conidial production, conidial germination or pathogenicity compared to parental isolates. Genetic transformation verified that the variation in the *MoIRR* gene was associated with resistance to IPT. This is the first report of a Zn_2_Cys_6_ transcription factor related to IPT resistance.

MoIRR is a Zn_2_Cys_6_ transcription factor since it has the conserved GAL4-like and Fungal_TF_MHR domains of well-studied Zn_2_Cys_6_ transcription factors, and it shared the highest sequence similarity with a non-annotated homolog in *Phaeoacremonium minimum*. Zn_2_Cys_6_ transcription factors have been well-studied in ascomycete fungi (Burger et al., 1991; Gómez et al., 2002). The six-cysteine-residue DNA-binding domain (DBD) located at the N terminal region of the protein is usually important for binding to the promoter of the regulated gene (Burger et al., 1991). In addition to the DBD domain, there are also regulatory domains such as the middle homology region (MHR) and activator domain at the C-terminal region. The Fungal_TF_MHR domain is very common to and exclusive in Zn_2_Cys_6_ transcription factors, and MHR may assist the C_6_ zinc cluster in DNA target discrimination (Schjerling and Holmberg, 1996). Future work should further identify promoter regions that interact with MoIRR using methods such as the yeast one-hybrid system that would shed light on the regulation network of MoIRR.

Generally, point mutations in essential or very important target genes may confer fungicide resistance; however, there were no observed fitness costs in *MoIRR* knockout transformants, and thus MoIRR is a less likely target for IPT. Because all IPT resistant isolates examined were also IBP resistant, by multidrug resistance (MDR) may be involved. Up regulated ABC or MFS transporters are usually involved in MDR. For instance, fluconazole resistance in *Candida dubliniensis* was caused by the overexpression of MDR1, a gene encoding an MFS transporter. In addition, the regulator of MDR1 (Mrr1) also contains a Zn_2_Cys_6_ domain in the N-terminal region (Schubert et al., 2011). The ascomycetous fungus *Botrytis cinerea*, the causal agent of gray mold of grapes, has also developed MDR based on a Mrr1 mutation which caused overexpression of the ABC transporter atrB (Kretschmer et al., 2009). The *PDR5* gene, conferring multidrug resistance, is positively regulated by the Zn_2_Cys_6_ transcription factors Pdr1p and Pdr3p, which are controlled by the repressor Rdr1.The ΔRdr1p strains show overexpression of the *PDR5* gene and exhibit increased resistance to cycloheximide (Hellauer et al., 2002). Unexpectedly, in the current study, no MDR was observed in 1a_mut, 1c_mut or ΔMoIRR transformants after amended agar tests with azoxystrobin, tebuconazole, carbendazim, boscalid, or cycloheximide (data not shown). Unlike Mrr1, which is a transcription activator for MDR, MoIRR negatively regulated resistance similar to Rdr1. The MoIRR-C-2 is more sensitive to IPT than sensitive parental isolate H08-1a implying that MoIRR might be a transcription repressor like Rdr1. Greater expression of MoIRR was also observed in MoIRR-C-2 than in H08-1a, which might be caused by more than one copy of MoIRR gene inserted in complemented transformants, but this remains to be tested. Because the *MoIRR* gene did not trigger MDR, this implies that the IPT resistance mechanism may be a novel mechanism of fungicide resistance.

In this study, IPT- resistant mutants and transformants showed cross-resistance to IBP, which was consistent with a previous research, suggesting that MoIRR is not only related to IPT resistance but also related to CBI resistance (Uesugi and Sisler, 1978). Based on the results observed in this study, various possible mechanisms could confer the resistance to IPT and IBP in *M. oryzae*. For example, *M. oryzae* could activate IPT- or IBP-specific efflux pumps, it could inhibit pathways that provide an alternative to choline biosynthesis, or it could inhibit pathways that perform general cell damage correction (i.e., chaperones, DNA repair, etc.) (Shapiro et al., 2012; Belenky et al., 2013).

In conclusion, the Zn_2_Cys_6_ transcription factor MoIRR has been demonstrated to be responsible for IPT resistance in *M. oryzae*, and MoIRR containing a typical Fungal_TF_MHR domain was found to be a core element associated with IPT resistance. Uncovering downstream pathways regulated by MoIRR would help to better understand the mechanism of CBI resistance. RNA-Seq analysis of differences between resistant mutants and the parental wild type isolate was considered an efficient way to target them. Meanwhile ChIP-Seq also had the advantage to find downstream pathways by directly targeting their promoter. Further work should be done to demonstrate how MoIRR regulates the downstream pathways for resistance to IPT, and provide a basis for further research into such resistance.

## Author contributions

C-XL and Z-QW conceived and designed the experiments. Z-QW, F-ZM and M-MZ performed the experiments. Z-QW, L-FY, W-XY, and YL analyzed the data. Z-QW and C-XL wrote the paper with help from TH, Y-LP and Z-HW. C-XL supervised the study. All authors read and approved the final manuscript.

### Conflict of interest statement

The authors declare that the research was conducted in the absence of any commercial or financial relationships that could be construed as a potential conflict of interest.
