# Supplementary material for: A Putative Zn2Cys6 Transcription Factor Is Associated With Isoprothiolane Resistance in Magnaporthe oryzae
Source: Front Microbiol. 2018 Oct 31;9:2608. doi: 10.3389/fmicb.2018.02608 (PMC6220061; doi:10.3389/fmicb.2018.02608)
Supplement: Supplementary file 1 [file Table_1.DOC]

**Table S1**. Primers used in this study

| **Primer** | **Sequence（5'to 3'）** | **Description** |
| --- | --- | --- |
| MoIRR/5'for | GCATAGATACACGGCGACAAG | primer for disruption DJ PCR |
| MoIRR/5'rev | TCAATATCATCTTCTGTCGATCTGAACCGTTATGGGTCTGG | same as MoIRR/5'for |
| MoIRR/5'nest | TGCTTTGCCTTTGCGTGTT | same as MoIRR/5'for |
| MoIRR/3'for | AGATGCCGGATCCACTTAACTTTGTTCTTGTGGCGTTGTTT | same as MoIRR/5'for |
| MoIRR/3'rev | CGTTTCATTTATGGTCTGGGTG | same as MoIRR/5'for |
| MoIRR/3'nest | GACGGGAGTCAGTGGAGTAGC | same as MoIRR/5'for |
| H2 | GGTAAATAGCTGCGCCGATGGTT | same as MoIRR/5'for |
| H3 | GAACATCGCCTCGCTCCAGTCA | same as MoIRR/5'for |
| hyg For | TCGACAGAAGATGATATTGAAGGAG | HYG amplification |
| hyg Rev | GTTAAGTGGATCCGGCATCT | same as hyg rev |
| Hyg-V | CCTAAATGAACCATCTTGTCAAACGAC | disruption mutant identification |
| IRR-V | GAGGAGGTCTGCGTTCGTAT | disruption mutant identification |
| MoIRR_RT_F | ATTCGCATCGGCTTCCT | disruption mutant RT-PCR identification |
| MoIRR_RT_R | ACCTGTGGCGTGATACCC | disruption mutant RT-PCR identification |
| G418+F | ATCCCCCTAGGTCGACGCCAGCAGT | complementary strain construction |
| G418-R | TCGCGGCTTCGAATCGT | same as G418+F |
| MoIRR/5'C_for18T(HindIII) | CCCAAGCTTGGCATGAATCCCCTACTTAATAGTC | same as G418+F |
| MoIRR/5'C_rev18T(SalI) | ACGCGTCGACCAAGACGCCAGATTCACAAAC | same as G418+F |
| MoIRR/5'R_for | GGCATGAATCCCCTACTTAATAGTC | replacement strain construction |
| MoIRR/5'R_rev | TCAATATCATCTTCTGTCGACAAGACGCCAGATTCACAAAC | same as MoIRR/5'R_for |
| MoIRR/3'R_for | AGATGCCGGATCCACTTAACTTTCACATGAATGTTGGTCGTT | same as MoIRR/5'R_for |
| MoIRR/3'R_rev | TGAACAGAGCCATCAGGGAA | same as MoIRR/5'R_for |
| MoIRR/3'R_rev | AAGGGCAGCGAAGTGGC | same as MoIRR/5'R_for |
| MoIRR976C>T-F | TCGCATCGGCTTCCTCA | point mutation sequence verification |
| MoIRR976C>T-R | TTCCCTCATGTTCCATCCTTT | same as MoIRR976C>T-F |
| beta-tubulin-qRTp1 | GTGACCCTCGCAACGGAAAG | MoIRR expression dectection |
| beta-tubulin-qRTp2 | CGACGAACTGGATGCTACGC | same as beta-tubulin-qRTp1 |
| MoIRR_qF | TCGTCCACTATCTTATCTC | same as beta-tubulin-qRTp1 |
| MoIRR_qR | GCTATCCTTATTACCACAC | same as beta-tubulin-qRTp1 |
|  |  |  |

**Table S2. Reliable variants in 1a_mut.**

| **Gene^1^** | **Contig location^2^** | **Position^3^** | **Origin^4^** | **Alteration** | **Variation type^6^** | **Variant impact^7^** |
| --- | --- | --- | --- | --- | --- | --- |
| hypothetical protein (MGG_12580) | scaffold76 | 356 | T | C | missense | Moderate |
| hypothetical protein (MGG_07027) | scaffold80 | 480454 | C | T | missense | Moderate |
| **hypothetical protein (MGG_04843)^8^** | **scaffold96** | **914432** | **G** | **A** | **missense** | **Moderate** |
| arrestin domain-containing protein (MGG_01045) | scaffold672 | 23472 | C | T | missense | Moderate |
| hypothetical protein (MGG_01668) | scaffold888 | 118022 | G | 19 bp^5^ | inframe | Moderate |
| hypothetical protein (MGG_08441) | scaffold1262 | 2914 | G | A | missense | Moderate |
| hypothetical protein (MGG_05722) | scaffold1090 | 499238 | T | C | missense | Moderate |
| averantin oxidoreductase (MGG_02982) | scaffold1308 | 27306 | 59 bp | C | frameshift | High |
| hypothetical protein (MGG_04332) | scaffold1310 | 2372 | T | TA | frameshift | High |
| hypothetical protein (MGG_10705) | scaffold1459 | 81016 | A | 19 bp | disruptive | Moderate |
| polyketide synthase (MGG_15097) | C173370 | 4385 | C | T | missense | Moderate |

^1^ Genes SNP located.

^2^ Contig SNP located.

^3^ Position located in contig.

^4^ SNP nucleotide sequence in reference genome.

^5^ Deletion or insertion variant, number represents fragment size.

^6^ Variations cause different type of effect.

^7^ Assessment of the putative impact of the variant.

^8^ Point mutation of MGG_04843 was also detected from 1c_mut.

**Table S3. Reliable variants in 1c_mut^1^.**

| **Gene** | **Contig location** | **Position** | **Origin** | **Alteration** | **Variation type** | **Variant impact** |
| --- | --- | --- | --- | --- | --- | --- |
| hypothetical protein (MGG_08092) | scaffold34 | 517314 | C | T | missense | Moderate |
| STE/STE20/PAKA protein kinase (MGG_06320) | scaffold513 | 158362 | C | T | missense | Moderate |
| FS4761 external transcribed spacer and 18S ribosomal | scaffold623 | 121 | G | 9 bps | frameshift | High |
| **hypothetical protein (MGG_04843)^1^** | **scaffold828** | **8937** | **C** | **T** | **missense** | **Moderate** |
| hypothetical protein (MGG_15234) | scaffold885 | 196880 | G | 6 bps | frameshift | High |
| Not annotated | scaffold920 | 1376 | 22 bps | C | chromosome_number | High |
| hypothetical protein (MGG_12094) | scaffold975 | 152279 | C | A | missense | Moderate |
| hypothetical protein (MGG_11269) | scaffold1060 | 240203 | T | C | missense | Moderate |
| Not annotated | scaffold1078 | 337 | 89 bps | T | chromosome_number | High |
| Not annotated | scaffold1165 | 679 | 55 bps | T | chromosome_number | High |
| para-nitrobenzyl esterase (MGG_15428) | scaffold1216 | 5021 | A | 12 bps | frameshift | High |
| sarcosine oxidase (MGG_10800) | scaffold1291 | 8739 | C | T | missense | Moderate |

All terms are same in Table S1.

^1^ Point mutation of MGG_04843 was also detected from 1a_mut.

**Table S4. Growth inhibition of wild-type and resistant mutants under 50 μg/ml** tricyclazole.

| **Isolates** | **Growth inhibition** | |  | |
| --- | --- | --- | --- | --- |
| H08-1a | 0.11 | 1a_mut | | 0.12 |
| H08-1c | 0.19 | 1c_mut | | 0.24 |
| H08-6c | 0.21 | 6c_mut | | 0.26 |
| **Wild type isolates** | **0.17±0.05a** | **Resistant mutants** | | **0.2±0.07a** |
| **P = 0.327^1^** |  |  | |  |

^1^P- value was conducted based on LSD test analysis indicate statistically significant differences.

A B


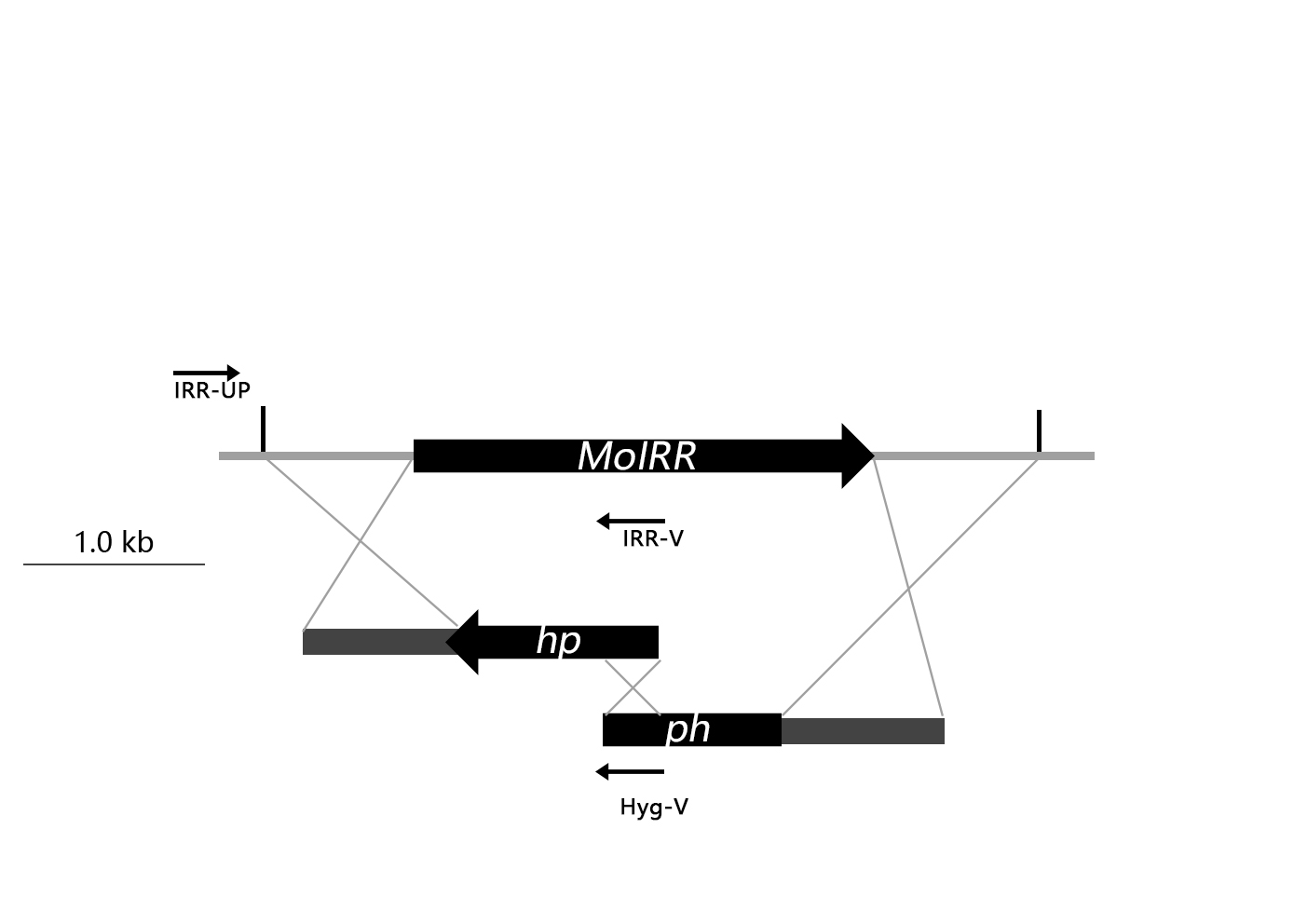

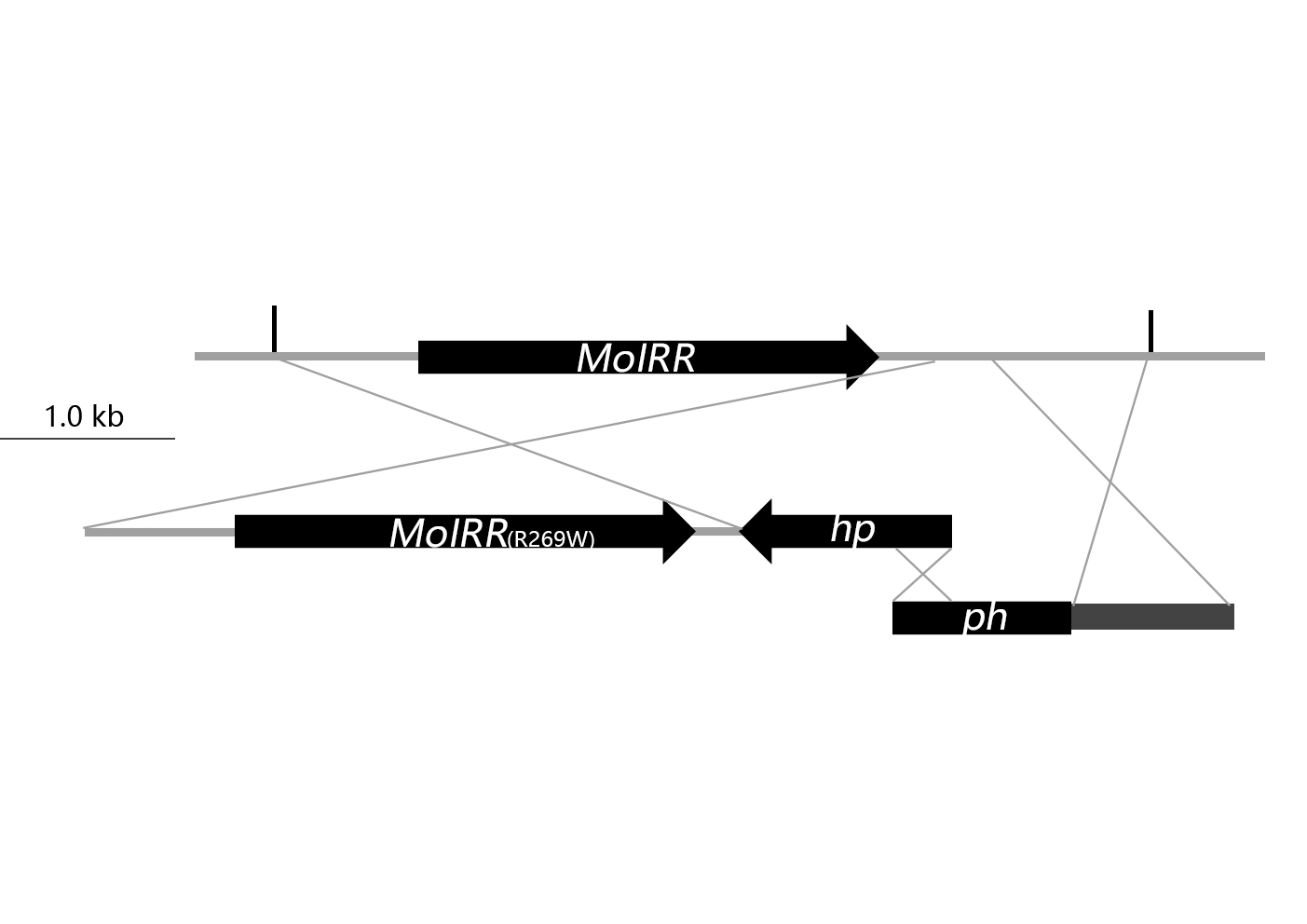


**Figure S1. Generation and identification of *MoIRR* knock-out and replacement mutants.**

(A) Strategic map of gene knock-out in sensitive isolate H08-1a. The upper part represents the genomic locus target of *MoIRR* gene. Upstream and downstream flanks were fused with partial hygromycin resistance cassette. Primer binding sites are indicated by arrows.

(B) Strategic map of *MoIRR* gene replacement in sensitive isolate H08-1a from 1a_mut with R269W.


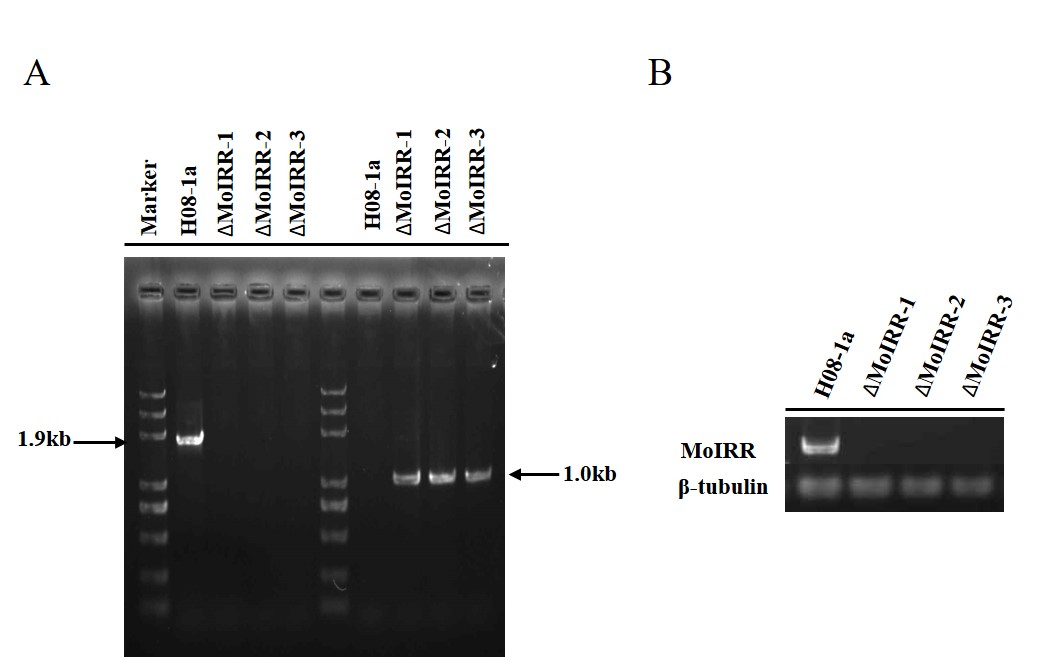


**Figure S2. Knock-out transformants were confirmed by specific PCR and RT-PCR**

A, PCR performed with primer pair IRR-UP/IRR-V with gDNA in left part, a 1.9-kb amplified fragment, indicates *MoIRR* presence. PCR performed with primer pair IRR-UP/Hyg-V with gDNA in right part, a 1.0-kb amplified fragment, indicates *MoIRR* replacement by hygromycin resistance hygromycin cassette.

B, RT-PCR was deployed to confirm expression level of *MoIRR* in knock-out transformants


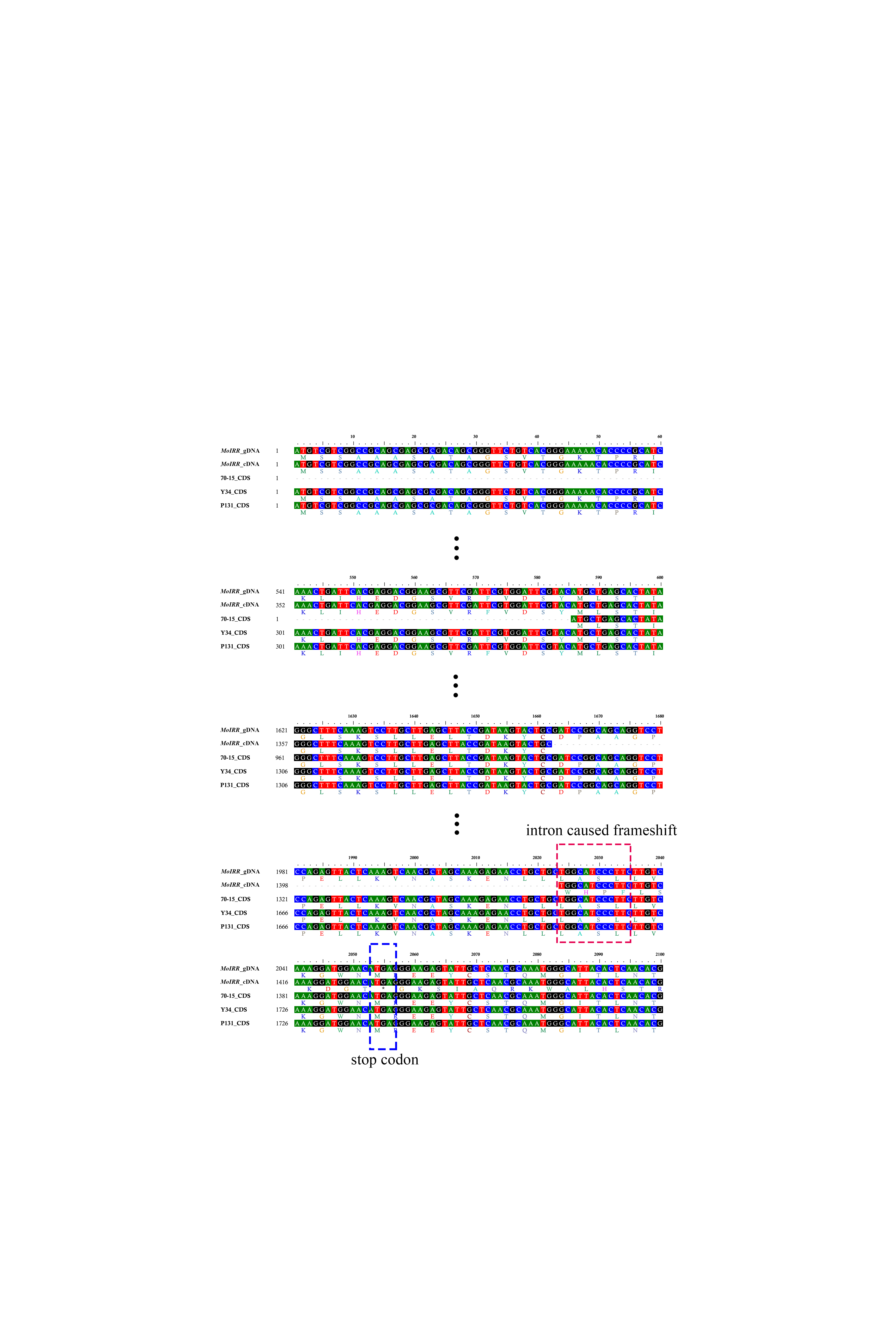


**Figure S3. *MoIRR* gene’s structure definition.**

*MoIRR* gene structure definition was investigated by comparing cDNA sequence with different version of predicted coding sequences from three published genomes. The last intron in *MoIRR* shifted the translation frame and stop translation at coding sequence position 1431, and encoding a protein with 476 amino acids. The actual transcript was much shorter than previously predicted in other published genomes. (*MoIRR*_gDNA was the whole gene sequence predicted in genome Y34. *MoIRR*_cDNA was the gene sequence amplified from H08-1a cDNA. 70-15_CDS was the coding sequence of *MoIRR* predicted in published genome 70-15, and accession number was XP_003712320. Y34_CDS was the coding sequence predicted in published genome Y34, and accession number was ELQ34729.1. P131_CDS was the coding sequence predicted in published genome P131, and accession number was ELQ59098.1).

**Figure S4. Coding sequence of MoIRR (accession number MH886651)**

**>*MoIRR***

**ATGTCGTCGGCCGCAGCGAGCGCGACAGCGGGTTCTGTCACGGGAAAAACACCCCGCATCCTCGCCTGCGTGCTGTGCCAGCATCGCAAAATAAAATGTGACAGATCTTTTCCGTGTGCGAATTGTCAGAGGGCTAACGTACAATGTACCCCGAGCACGCCCGCGCCTGCGCGCAAGCGAAGAAGACCAAACCAGGACCTTCAGGAGAGATTGGCGCGTTGCGAGGAACTATTGAAGGAATACGCACCAGATGGCAAGCCCGACATCGCAAGACTGACTACGAAACGCTCCAGCTTATCCCAGAGTCCACCCAAGGGCGAGGAGCCGTTGCCAGAATGGAACCGCCACGGCAAACTGATTCACGAGGACGGAAGCGTTCGATTCGTGGATTCGTACATGCTGAGCACTATATATGACGAGCTTCGGGCCATGCGTGACATCATTGACCATGACGAGAGCACCCCGGAAGACGAAAGCTCCGATTACATGACCCCGGATACGAACGCAGACCTCCTCTTTGGCGGAGATACTCCGCAACCCGCAGGAAACTCCATGGAATTGCAACCAAGTCCAGGGCACATCTTCCGCTTGTGGCAGGTGTTCCTGGATCGGGTCAACCCCCTAATCAAGCTCGTCCATGTACCGTCTCTCCAACCATATTTTGTTGAAGCTACTGCCGGTGCCCCGCTGCCAAAGAATATCGAGGCCTTGCTGTTCTCCATCTATACGCTCGCCGCTGTCTCGTTGTCCGATGCAGAATGCACATCTATACTCGGATACGGCCGGGAGGCGGCCCTCCATCGATTTTCTTCGGGTGTTCGCGCCAGTCTAATTCGCATCGGCTTCCTCAAAACACACGATTTGACCACGCTTCAAGCGCTCGTCCACTATCTTATCTCGCTGCAGGGCAGATACAACAGGCATGCAGCATGGGTGCTCAATGGTGTGGTAATAAGGATAGCTCAGAAGATGGGCATACATCGGGACGGCACAATGCTGGGTCTACCGCCTTTCGAGACTGAGATGCGGCGACGTCTTTGGTTTCAAATATTGTCAATGGAGTTCAAGACTGCTCTTATGTCCGGcCTAGGCCATTCACTACTCCCAAGAGTCTGGGACACCCAAGAACCCAAGAACGTCAACGATGCAGATCTGCATCCCTCAGCGACCGAGCCCGTCAAAGACCGAGAAGGCCCGACCGAGATGATTTTTGTTCTGATCACGAACAAAGTTGCCCGTTTCATCGTGGAAAGTCCCGGAATTGAGCCTATTTTCCTGTACCACGACGAGAAGGTCAAAAAAATACCAGGCGCACCAAGCGAGGAAAAGATAAAAGAGTTTCGCGGCCTGATTGATGGGCTTTCAAAGTCCTTGCTTGAGCTTACCGATAAGTACTGCTGGCATCCCTTCTTGTCAAAGGATGGAACATGA**
